# Adult male circumcision in Nyanza, Kenya at scale: the cost and efficiency of alternative service delivery modes

**DOI:** 10.1186/1472-6963-14-31

**Published:** 2014-01-23

**Authors:** Elliot Marseille, James G Kahn, Sharone Beatty, Moguche Jared, Paul Perchal

**Affiliations:** 1 Health Strategies International, 555 59th St., Oakland, CA, 94609, USA; 2 Philip R. Lee Institute for Health Policy Studies, University of California, San Francisco, USA; 3 EngenderHealth, 440 Ninth Avenue, New York, NY, 10001, USA; 4 EngenderHealth, Kisumu, Nyansa, Kenya

**Keywords:** Adult male circumcision, Cost, Efficiency, Service delivery, Cost-effectiveness, HIV prevention

## Abstract

**Background:**

Adult male circumcision (MC) services in Kenya are provided through both horizontal and vertical programs, and via facility-based, mobile and outreach service delivery. This study assesses the costs and composition of unit costs for each program approach and service delivery mode and assess the cost-effectiveness of each.

**Methods:**

This study was conducted on the unit costs of adult MC delivery in 222 purposively-selected MC delivery sites in Nyanza Province, Kenya from November 2008 through April 2010 using program data from the AIDS, Population, and Health Integrated Assistance Project II (APHIA II) and from the Nyanza Reproductive Health Society (NRHS). The former program can be characterized as horizontal or integrated; the latter as ‘diagonal’; containing both horizontal and vertical elements. Expenditure and services data were collected from project financial and monitoring documents and via discussions with program officials. In addition, per-case, direct service delivery costs were calculated using time and motion observations of 246 adult MC procedures performed during May and June, 2010. We calculated the cost per HIV infections averted for each of the service delivery modalities.

**Results:**

Unit cost per adult MC was $38.62 and $44.24 for APHIA II and NRHS respectively, ranging from $29.32 (APHIA II mobile) to $46.20 (NRHS outreach/mobile). Unit costs at base facilities was similar for the two approaches. Time and motion data revealed that the opportunity cost of the elapsed time between the arrival of the surgical team and the time the first MC procedure begins varies between $2.08 and $6.27 per case. The cost per HIV infection (HIA) averted ranged from $117.29 for mobile service via the horizontal APHIA-II program to $184.84 per HIA for the diagonal NRHS program.

**Conclusions:**

This study provides evidence for the similar efficiency of a horizontal approach (APHIA II) and a combination of horizontal and vertical approaches (NRHS) to support scale-up of adult MC services in Nyanza. Differences in unit cost are modest, not consistently in the same direction, and largely explained by differences in compensation levels.

## Background

The efficacy, effectiveness and cost-effectiveness of adult male circumcision (MC) to prevent female-to-male transmission of HIV are well established [1-6]. The World Health Organization (WHO) estimates that as many as 2 million new infections in Sub-Saharan Africa can be averted in the next 10 years through scale-up of safe, high-quality MC services [7].

HIV prevalence in Kenya in 2012 was more than five times higher among uncircumcised men than among circumcised men ages 15–64 (16.9% vs. 3.1%) [8]. In Nyansa Province, based on 2009 data, the equivalent figure were 17.3% and 5.5% respectively [9]. Among Kenya’s nine provinces, Nyansa has the highest combined male and female HIV prevalence, 13.9%. Nyanza accounts for one in three new infections in Kenya [10], while comprising only 5.4 million of Kenya’s 2009 population of 38.6 million [11] making it a priority region for scaling up MC.

In 2009 the Kenya Ministry of Health (MoH) released the *Kenya National Strategy for Voluntary Medical Male Circumcision*[12] to guide scale-up of MC. Given system constraints, fixed health care facilities are not equipped to meet short-term MC strategic targets. The national strategy therefore supports adult MC service delivery through mobile services in addition to fixed facilities. A number of international and local NGOs, have supported the rollout of the national MC program in Nyanza through a combination of horizontal and vertical approaches. The AIDS, Population, and Health Integrated Assistance Project II (APHIA II), funded by the U.S. Agency for International Development (USAID), has supported horizontal service delivery in which non-dedicated MoH teams, working an average of 12–38% of a 220-day work-year, provide adult MC services integrated with routine health services at base facilities, outreach sites, and mobile locations. Nyanza Reproductive Health Society (NRHS), funded by the U.S. Centers for Disease Control and Prevention (CDC), supports “diagonal” service delivery (a combination of horizontal and vertical program approaches). In this approach, dedicated MC teams employed by NRHS work 100% of a 220-day work-year, providing adult MC services at base health facilities, outreach sites and mobile locations (the vertical aspect), while also supporting non-dedicated MoH staff to integrate MC services (the horizontal aspect) into the provincial health care system. APHIA II supported the MoH implementation of MC services from October 2008 to October 2010, and NRHS has supported the MoH since October 2008.

Few studies in Kenya or elsewhere have compared the cost and efficiency of different MC delivery modes [4,13,14]. This study, conducted from May 2010 to April 2011, assesses the cost of delivering adult MC services in Nyanza Province, Kenya. It aims to assist planning for adult MC scale-up by addressing three questions: 1) what is the current cost per MC, and how does this unit cost or “efficiency” vary by program approach (horizontal versus diagonal) and service delivery mode (base facilities, outreach services, and mobile services)? 2) what is the composition of these unit costs for each program approach and service delivery mode; and 3) what is the cost per infection averted (HIA), that is “cost-effectiveness” for MCs and how do these vary by modality? In an appendix we explore strategies for increasing efficiency that are suggested by these cost findings.

## Methods

### Description of adult MC program approaches and service delivery modes

We analyzed costs of adult MC services implemented by APHIA II and NRHS in collaboration with the MoH, in accordance with the National Strategy for Voluntary Male Circumcision. Table 1 describes the key elements of each approach. In both, MC is provided as part of comprehensive HIV prevention package that includes correct and consistent use of condoms, reduction in the number of sexual partners, delay in the onset of sexual relations, treatment of sexually transmitted infections (STIs) and HIV counseling and testing. Surgical teams are composed of a surgeon and surgical assistant, a counselor, and an infection prevention specialist. Due to task shifting and task sharing, various cadres of personnel play more than one role depending on the circumstances. For instance, a trained nurse may be a surgeon, an assistant or even a counselor. In general however, “Surgeons” were Clinical Officers, and occasionally Nurses or Medical Officers; “Surgical Assistants” are Nurses and occasionally Clinical Officers; “Counselors” are Counselors or nurses; and “Infection Prevention Specialists” are Hygiene Officers. Surgery was conducted by the forceps-guided method using re-usable surgical instruments.

**Table 1:** Key elements by program approach

**Program approach**	**Key elements**
**Horizontal**	○ Non-dedicated MoH MC teams, working an average of 12-38%* of a 220 day work year, provide MC services integrated with routine health services.
	○ Teams are based at, and provide services at, district or sub-district hospitals as well as at outreach and mobile locations.
	○ Additional strategies used for meeting demand during high volume periods, such as the Rapid Results Initiative**, include task shifting to nurses, scheduling more MC services during evenings and weekends, and ensuring a sufficient number of non-dedicated MoH MC teams are scheduled to provide adequate coverage to meet demand.
	○ APHIA-II provided the MoH with the following types of technical assistance:
	▪ Minor renovations to surgical theaters
	▪ MC supplies/equipment
	▪ Training and supportive supervision
	▪ Quality assurance
	▪ Client flow optimization
	▪ Vehicles for transporting outreach and mobile teams
	▪ Demand generation through collaborating with public health officers to carry out one-on-one and group mobilization strategies
	▪ Community engagement activities
**Diagonal** (Combination of Horizontal and Vertical)	○ Dedicated MC teams employed by NRHS, working 100% of a 220 day work year, provide MC services at base health facilities, outreach sites, and mobile locations to supplement MC services offered by the MoH.
	○ Teams are based at the NRHS office in Kisumu and travel to base facilities as well as outreach and mobile locations.
	○ Additional strategies used for meeting demand during high volume periods, such as the Rapid Results Initiative, include task shifting to nurses, hiring short term contract staff, and scheduling more MC services during evenings and weekends.
	○ NRHS provided the MoH with the following types of technical assistance:
	▪ Minor renovations to surgical theaters
	▪ MC supplies/equipment
	▪ Operating a MC training center for certifying MC providers
	▪ Quality assurance
	▪ Supportive supervision
	▪ Client flow optimization
	▪ Vehicles for transporting outreach and mobile teams
	▪ Demand generation through one-on-one and group mobilization strategies
	▪ Community engagement activities

*Estimated based on the number of MCs performed per day during the study period.

**RRI is a strategy used by Government Ministries and Departments to tackle large scale change efforts through a series of small-scale, result-producing and momentum building initiatives. The Government of Kenya applied the RRI approach to MC from November to December 2009, which coincided with the school holidays.

In addition to adult MC services provided at base facilities on an ongoing basis, outreach and mobile delivery, are being implemented by the APHIA II and NRHS to improve access and coverage of MC services at the community level. In both the outreach and mobile modes, health workers travel to clinics, dispensaries, and other community locations to provide MC services on a periodic and temporary basis, typically 1–2 days. Table 2 summarizes the key features of the three service delivery modes we assessed.

**Table 2:** Key features of MC service delivery modes

**Service delivery mode**	**Key Features**
**Base**	○ A district or subdistrict hospital in an urban or semi-urban setting provides ongoing MC services.
	○ The facility meets standard MC surgery requirements (e.g., has trained staff, supplies, surgical instruments, an appropriate space).
	○ In the case of base sites supported by NRHS, MC procedures are supplemented by NRHS dedicated MC teams; APHIA II base sites rely on existing MoH staff to provide MC services.
**Outreach**	○ A health center or dispensary in a rural setting that is not staffed/equipped to provide routine MC; receives supplemental inputs (e.g., trained MC surgeons/surgical assistants, equipment, surgical instruments, supplies, transport) from a “base” facility to provide MCs that meet standard MC surgery requirements during prescheduled MC days.
	○ The receiving facility contributes minimal or no inputs (e.g., local technical support, supplies) other than providing a space for surgeries.
**Mobile**	○ A fully contained MC surgical unit (consisting of a trained MC surgeon/surgical assistants, equipment, surgical instruments, supplies, and transport) is able to stage MC procedures that meet standard MC surgery requirements at any location (e.g., a school, community center, tent, etc.), including remote settings.
	○ The receiving facility provides the space for surgeries only.

For NRHS, it was not possible to disaggregate cost information from the field into outreach versus mobile, so we list these combined activities under the term “outreach/mobile.” About 90% of the field-based MCs provided by NRHS are performed in an outreach setting; thus, the term “Outreach” is comparable for APHIA II and NRHS.

### Site selection and sample size

As shown in Table 3, a total of 222 service delivery sites were selected for the study. The 222 MC sites cover all MCs that were carried out during the study period. All were located in rural areas. Criteria for selection of those sites included adequate hygienic conditions, anticipated demand based on catchment size, and supply constraints such as the staffing and space available at the outreach clinics. The outreach and mobile data set contains information on MCs that were delivered during one multi-day visit per site.

**Table 3:** Number of study locations, by MC approach and service delivery mode

	**Base**	**Outreach**	**Mobile**	**Total**
APHIA II (horizontal)	3	28	13	**44**
NRHS (diagonal)	20	158		**178**
**Total**	23	199		**222**

For these locations, we collected comprehensive retrospective expenditure and services data from the MoH, from the EngenderHealth APHIA II Nyanza office, and from NRHS Nyanza office financial documents and discussions with program officials.

### Time and motion (T&M) observations

In May and June 2010, a member of the EngenderHealth research team, with knowledge of the service delivery team observed 246 adult MC procedures at 35 sites in six districts to collect information for a T&M analysis. 122 of the observed procedures were supported by APHIA-II and 124 by NRHS. The average age of patients was 20.8 years and 20.6 years of age, respectively. We conducted these observations to measure the staff resources required for MC, including the number of MCs performed per surgery-day; the time required for specific steps of the MC procedure and the waiting time prior to the first surgery of the day.

### Ethical approval

EngenderHealth obtained ethical approval for this research from the FHI 360 and Kenya Medical Research Institute institutional review boards. Concurrence from the Nyanza Ministry of Public Health and Sanitation through a memorandum of understanding ensured a collaborative partnership between this ministry and EngenderHealth. Written informed consent was obtained from clients, providers, and program officials prior to participation. Confidentiality was protected through secure data storage, including stripping the data of identifiers.

### Training and piloting

We used local data collectors and provided them with a seven-day training in Nyanza, in May 2010, including topics on research ethics and use of the study tools. The meeting was also used to provide input on the study tools before they were finalized.

### Analysis of cost per adult MC delivered, by service delivery mode

Total unit cost and incremental per-case cost defined as the average cost of providing each additional surgery-day divided by the cases performed were calculated through Excel-based costing instruments. Both total unit cost and the incremental per-case cost, were tabulated by cost component (e.g., personnel, supplies) and by service delivery mode. Costs were converted to US dollars at the rate of 76.6 shillings per dollar, the market rate in July 2009, mid-point of the unit cost study period (http://www.xe.com).

The number of MCs performed each month for each service mode were obtained from routine monitoring records at each site. The incremental per-case cost was the cost of staff members’ time for the procedure including set-up and waiting time, instrument sterilization, cost of each expendable supply item, and transportation to deliver outreach and mobile activities, including driver and staff time in transit divided by surgeries delivered. These resources were measured using a T&M instrument that lists the sequential activities needed to complete a forceps-guided MC adapted from WHO’s *Male Circumcision Models for Optimizing the Volume and Efficiency of Services*[15], and the start and stop times for each activity. We recorded the quantity and type of expendable supplies consumed, and obtained the market unit cost paid by APHIA II and NRHS from their procurement records.

#### Unit costs

The costs per MC delivered were calculated from November 2008 when significant MC services began, through April 2010, the most recent time for which expenditure data were available. Unit costs consist of direct variable costs, including personnel providing direct services, supplies, transport costs, demand generation activities; and indirect costs such as administrative support, training, facility rents and renovations, capital equipment, and utilities. Capital costs were amortized over five years, with no salvage value. Training was assumed to have a life of three years. Shared resources were allocated to MC activities via direct allocation methods [16] (see Additional file 1 for details on allocation methods).

### Cost-effectiveness and cost savings

We estimated that in Nyanza, with an HIV prevalence of 20%, 17% in uncircumcised men, 26% in women [9,17], 10 MCs avert about 2.5 HIV infections over 20 years [18], or 0.25 HIV infections averted per MC. This estimate includes indirectly averted future infections of female sex partners, discounted to the present at 3% per annum. Finally, we calculated a cost-effectiveness value, cost per HIV infection averted (HIA).

## Results

### Cost per adult MC delivered, by program approach and service delivery mode

Table 4 summarizes the number of adult MCs delivered, by approach and service delivery mode. During the study period, a total of 62,705 MCs were delivered, 90.1% through the NRHS diagonal approach. Overall, community-based services dominated the caseload, with 68.6% of MCs delivered at either mobile or outreach sites. APHIA II delivered 53.5% of its MCs at outreach or mobile sites, while NRHS delivered 70.3% of its MCs at outreach/mobile sites.

**Table 4:** Number of MCs delivered, November 2008–April 2010, by approach and service delivery mode

	**Base**	Outreach*	**Mobile**	**Total**	**% of total**
APHIA II	2,897	2,829	485	6,211	9.9%
NRHS	16,791	39,703	n/a	56,494	90.1%
Total	19,688	42,532	485	62,705	100.0%
% of total	31.4%	67.8%	0.8%	100.0%	

*For NRHS, “outreach” signifies combined outreach and mobile activities. Of these, about 90% took place at permanent facilities and 10% at mobile locations. The procedures were therefore predominantly “outreach” in nature.

**Figure 1:**
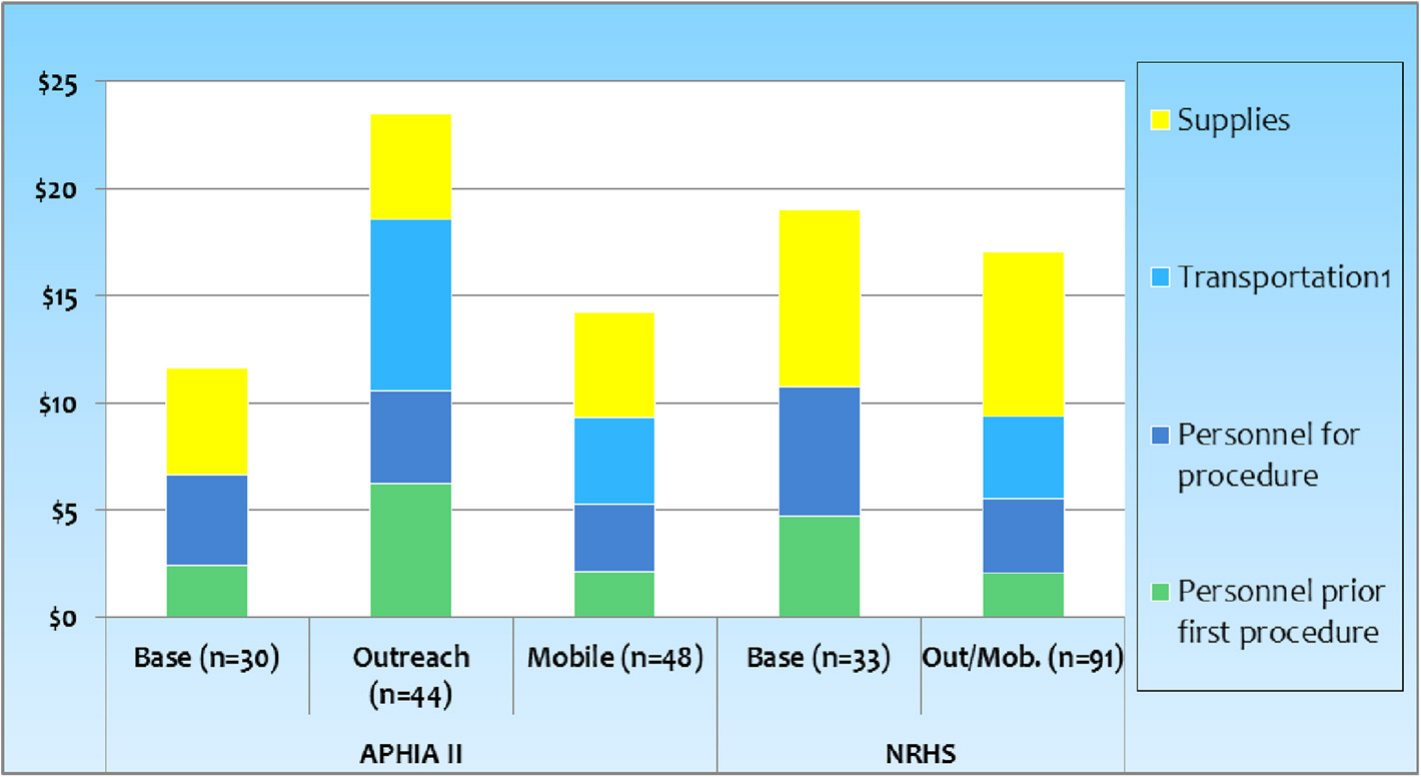
**Incremental per-case costs and their components, by service delivery mode and approach.***Note:* Costs reflect the resources required to provide a day of surgery and includes staff’s set-up and wait time. These estimates are derived from 246 T&M observations. *NRHS outreach includes approximately 10% mobile. †Transportation assumes $0.37 per km, including fuel, maintenance, depreciation, insurance (NRHS records); and compensation of personnel time in transit.

Figure 1 presents the incremental per-case costs of delivering a MC surgery. Incremental per-case costs were lowest for APHIA II at base facilities ($11.62), which have no transportation costs. Outreach services for the APHIA II approach had the highest incremental per-case cost ($23.52), due primarily to the cost of transportation and to a long average waiting time for the MC team prior to the first surgery of the day. The NRHS approach delivered MCs with a marginal cost of $19.06 at base facilities and $17.04 at outreach sites. The cost of personnel activity prior to the first procedure including wait time constituted between $2.08 (NRHS, Outreach/mobile) and $6.27 (APHIA II, Outreach), or between 12% and 27% for NRHS, Outreach/mobile and APHIA II, Outreach, respectively of the incremental cost per case.

**Figure 2:**
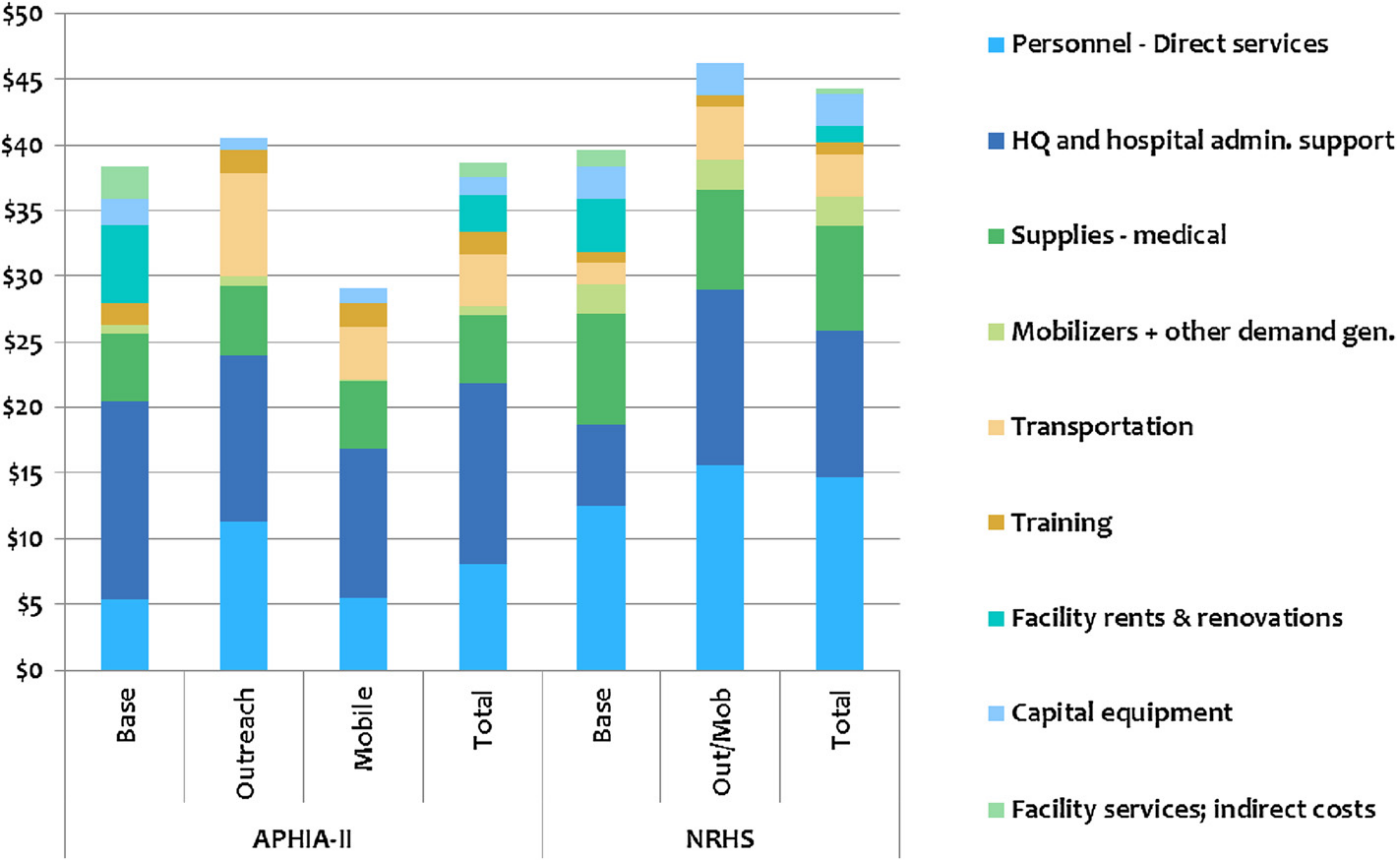
**Unit cost of MC provision, by agency and service delivery mode.***Note:* Transportation includes fuel, maintenance, depreciation, insurance, and the value of staff time.

For the APHIAII program as a whole, the cost per MC was $38.62, and for NRHS it was $44.24, a difference of 15.5%. (see Figure 2)The total cost per MC was lowest for APHIAII mobile services, $29.32, and was highest for NRHS outreach services, $46.20, a difference of 37%. (see Additional file 2 for the percentage distribution of cost across components).

### Time and motion analysis

#### Number of MCs performed per surgery-day

**Figure 3:**
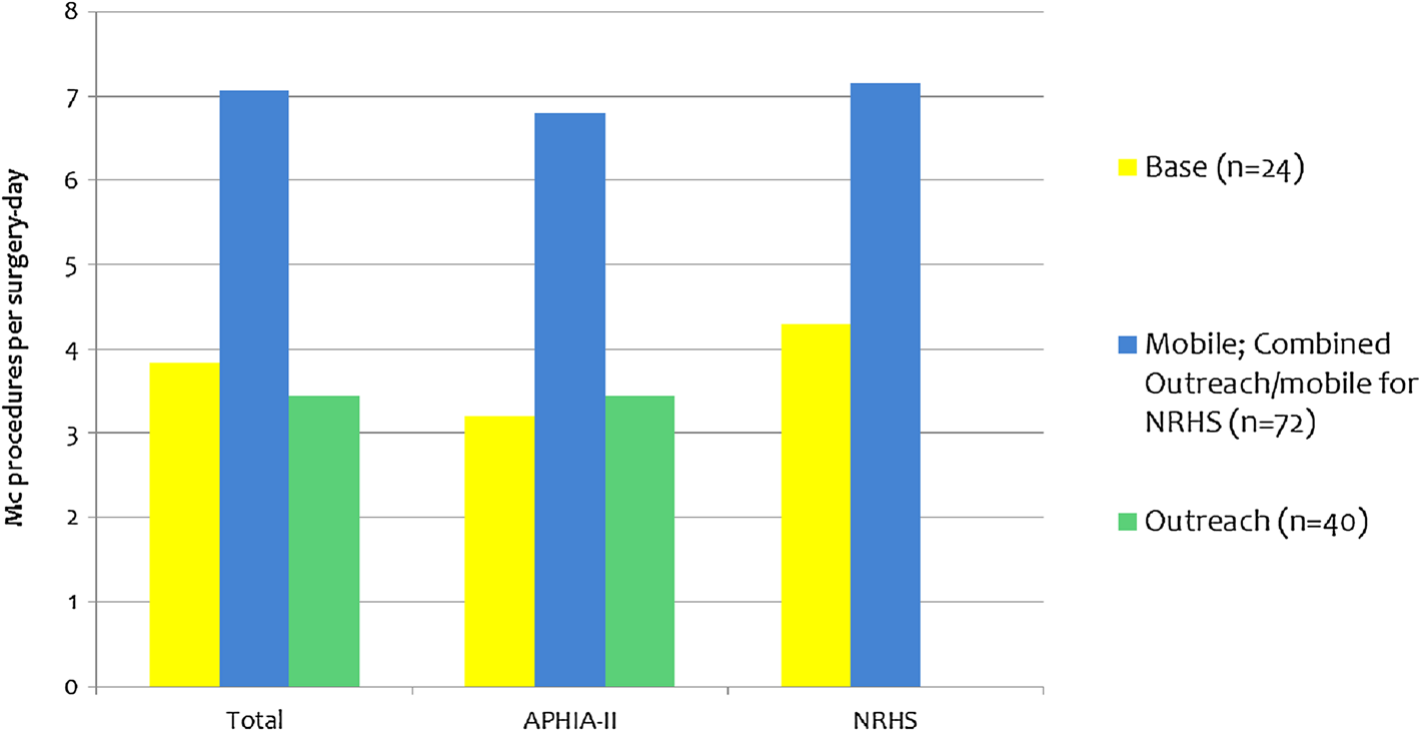
**Average number of MC procedures performed per surgery-day, by service delivery mode.***Note:* The n values represent surgery-days observed.

Figure 3 summarizes the number of adult MCs performed per surgery-day, based on 246 T&M observations. The number was lowest at the APHIA-II supported base facilities (3.2) and highest at the NRHS outreach/mobile sites (7.2).

#### Time required for the MC procedure

Table 5 shows the time required per adult MC for each service delivery mode. The average procedure time varied from 22.2 minutes (mobile) to 31.0 minutes (outreach), and total time (including postoperative time) varied from 23.3 minutes (mobile) to 32.9 minutes (outreach).

**Table 5:** Average time (in minutes) for MC case, by delivery mode

	**Base (n = 63)**	**Outreach (n = 43)**	**Mobile (combined outreach/mobile for NRHS) (n = 139)**	**Total**
Surgeon time (in minutes)	16.3	15.9	11.5	13.0
Procedure time (in minutes)	29.5	31.0	22.2	25.1
Total time per case (in minutes)	30.9	32.9	23.3	26.4
Surgeon time as % of total	53%	48%	49%	49%

*Note:* n = number of MC procedures observed.

On average, the cases performed through APHIA II required 6.1 minutes more than the NRHS cases, of which 2.7 more minutes were needed for steps requiring a surgeon’s active participation. These differences were statistically significant (p < 0.05). (see Additional files 3 and 4 for more comparisons of time per MC by approach and service delivery mode).

#### Waiting time prior to the first surgery of the day

**Figure 4:**
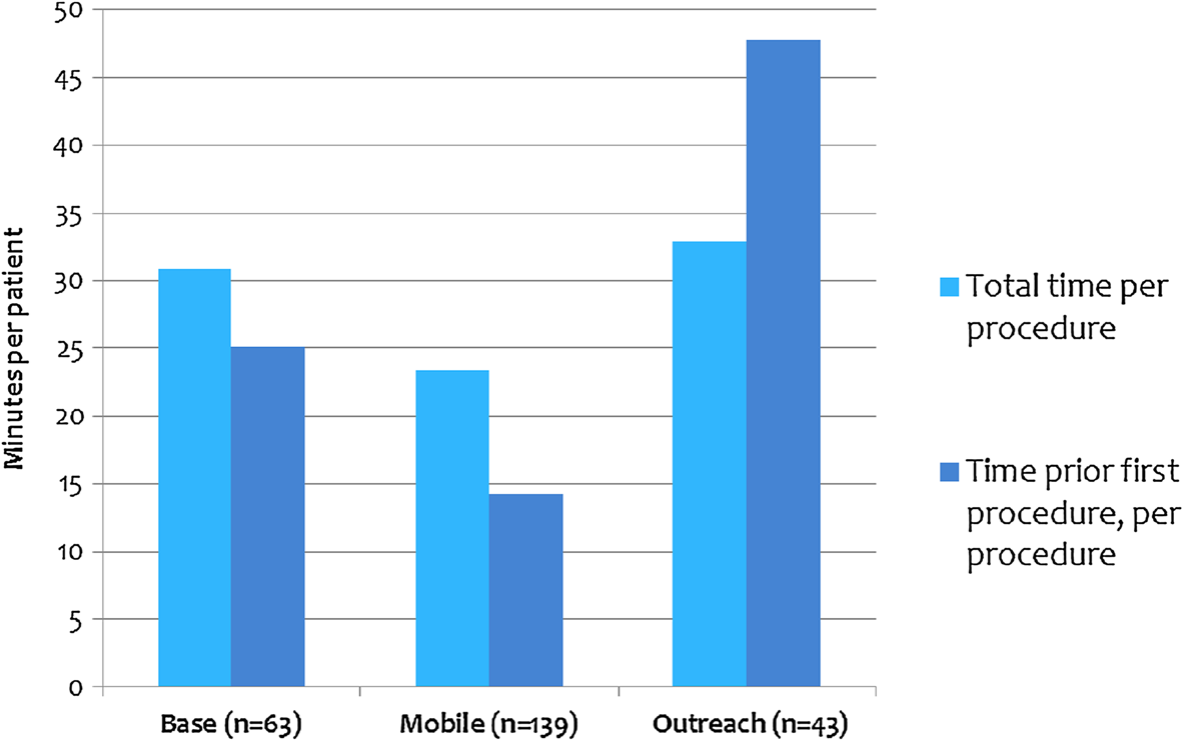
Total time and time before first MC of the day, per procedure, by delivery mode.

Analysis of T&M data found that a significant amount of time elapsed between the arrival of the surgical team and the time MC services began for the first patient. As shown in Figure 4, the waiting time varied from 48.8 minutes per case performed at outreach sites to 14.3 minutes per case performed at mobile sites.

At APHIA II-supported services, the waiting times varied from an average of 1.0 hours at base sites to 1.7 hours at mobile and 2.7 hours at outreach sites. At the NRHS-supported services, they varied from 1.6 hours for combined outreach/mobile sites to 1.9 hours at base.

### Relationship between cases per surgery-day and waiting time before first surgery

**Figure 5:**
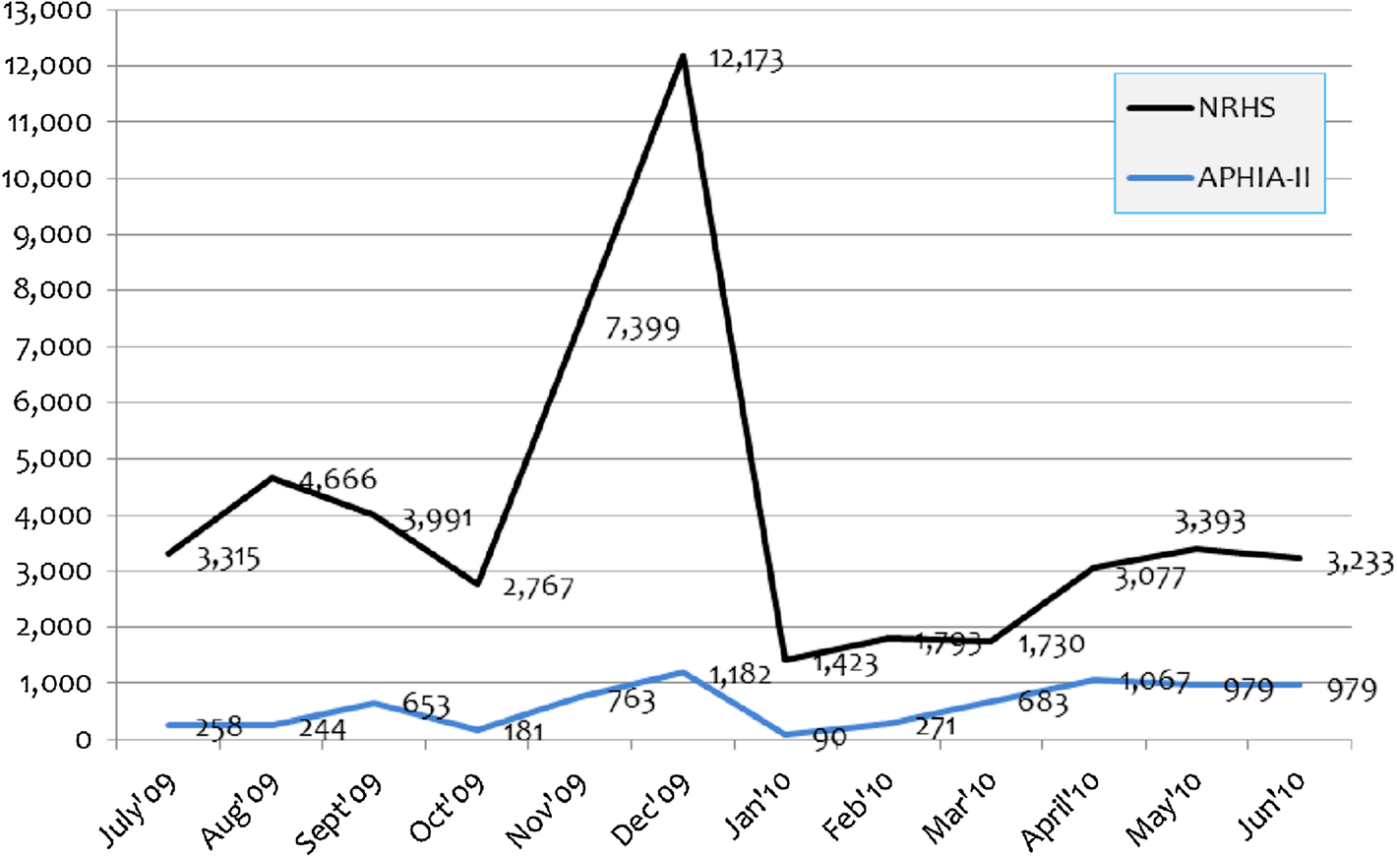
**Caseload trends for NRHS and APHIA II, July 2009–June 2010.***Note:* T&M data were collected in May and June 2010.

We found that the number of MCs per day was unrelated to waiting time at the start of the day (r = 0.016). However, when we disaggregated the data by approach, NRHS showed a substantial correlation between waiting time and the number of cases per day, (r = 0.37), while APHIA II showed no correlation (r = 0.04). Caseload trends for the 12-month period ending on June 30, 2010 indicate that the caseload for NRHS during the two-month period of T&M data collection (May and June, 2010) was much lower than the peak in December 2009 (see Figure 5).

It is possible that at higher caseload levels, waiting times were lower. However, for APHIA II, the T&M data were collected at a period of high case volume, not far below APHIA II's peak in December 2009. The available data thus provide substantial evidence that higher caseloads are not associated with shorter waiting times.

### Cost-effectiveness

As shown in Table 6, the unit cost of an adult MC surgery ranges from $29.32 to $46.20. The cost per HIV infection averted ranges from $117.29 (APHIA II, Mobile) to $184.82 (NRHS, Outreach/mobile). If implemented in a setting with half the incidence of Nyanza cost–effectiveness ranges from $234.58 to $316.64 per HIA. In settings with HIV incidence 50% higher than in Nyanza, the cost per HIA ranges from $78.18 to $108.02.

**Table 6:** Cost per MC and cost-effectiveness (US$ per HIV infection averted) of MC program, by delivery mode and agency type

		**APHIA-II**		**NRHS**	
	**Base**	**Outreach**	**Mobile**	**Base**	**Out/Mob**
Cost per MC	$38.33	$40.51	$29.32	$39.58	$46.20
If HIA per MC = 0.25 (base case)	$153.30	$162.03	$117.29	$158.32	$184.82
If HIA per MC = 0.125 (low incidence)	$306.60	$324.05	$234.58	$308.93	$316.64
If HIA per MC = 0.375 (high incidence)	$102.20	$108.02	$78.19	$102.98	$105.55

## Discussion

### Cost differences between program approaches

The most important finding of this study is that the unit cost differences between the horizontal and diagonal program approaches are modest, $38.62 for APHIA II and $44.62 for NRHS. Ninety percent of the adult MCs conducted during the study period were through the NRHS diagonal approach and 10% through the APHIA II horizontal approach. NRHS’ greater share of the total is due to the use of dedicated (full-time) MC teams that contrasts with non-dedicated (part-time) MoH teams providing MCs during only 12–38% of their time in the APHIA II approach. NRHS also deployed 2–9 dedicated teams per district, compared with 2–3 non-dedicated teams deployed by the MoH with APHIA II support. When the surgery sessions are underway, the teams work at capacity, but staff wait times, particularly before the start of the surgical sessions, are substantial. The unit cost figures we report include the cost of all MC-related staff time including wait time, and thus capture these inefficiencies.

The larger NRHS service volume may suggest that the diagonal NRHS approach can be scaled up more quickly in the short term and can increase service volumes over time. However, long-term operational challenges may emerge as it seeks to achieve fuller integration with existing MoH adult MC services. The horizontal APHIA II approach, while producing consistently lower service volumes, has also demonstrated rising service volume. The NRHS strategy may be more suited for rapid clearing of the unmet demand for MC, whereas the APHIA II strategy, since it has been more thoroughly integrated with the Kenyan health care system, may be more suited to serving the smaller volume of ongoing new cases. Of Kenya’s provinces, Nyanza experienced the highest increase in male circumcision rates, between the 2007 and 2012 Kenya AIDS Indicator Surveys from 48% in 2007 to 66% in 2012, and thus may approach this more routine caseload demand over the next few years.

Given the shortage of health workers in Nyanza across all cadres [19], shifting resources in the direction of either approach in the short term is no substitute for long-term investments to increase the health care workforce. Hiring should be done in a way that ensures the right balance of non-dedicated and dedicated MC workers. Disparities in health worker wages can potentially contribute to inequities through internal migration of health workers to MC from other important health services and from one geographic area to another. Disparities in wages may also contribute to a lack of motivation among existing MoH staff in the absence of MC-specific incentives. Decisions regarding which MC program approaches to emphasize should consider human resource shortages, cost, and long-term sustainability within the aims of national health policies and strategies.

### Unit costs by service delivery mode

In assessing unit costs by mode, a more complex picture emerges. Unit costs for adult MCs delivered at base facilities are very similar, $38.33 and $39.58 at APHIA II and NRHS, respectively. This is likely due to the fact that the majority of NRHS-supported base sites are MoH sites. The difference between outreach at APHIA II and combined outreach/mobile at NRHS sites is also modest, $40.51 vs. $46.20, a difference of 14.1%. However, the difference in unit cost between mobile MCs supported by APHIA II and outreach/mobile services supported by NRHS is more substantial, 36.5% higher in the latter. Part of this difference can be explained simply: Compensation for direct service providers at the APHIA II-supported sites is 45% of the level of equivalent staff compensation at NRHS. If the cost of direct service personnel at NRHS were reduced accordingly, the difference in unit costs for this portion of field activities drops to 22.1%, and the direction reverses: $40.51 at APHIA II–supported sites versus $37.61 at NRHS sites. Thus, it is hard to explain differences in unit costs by factors that are inherent in the relative virtues of a horizontal versus diagonal approach. On balance, we believe that higher efficiencies are more likely to be attained by adjusting the way MC activities are implemented within service delivery modes in either approach, than by attempting to select one broad approach as generally more efficient than the other.

Placing the results reported here in a broader context, the unit costs are of the same general magnitude as those reported elsewhere in the MC cost literature. The Futures Group has empirically estimated the unit cost of MCs in various African settings at $35–$50 [5]. Other modeling of adult MC scale-up in 16 geographic areas estimated an average of $168 per HIA and 5.6 MCs per HIA, thus implicitly $30 per MC [13]. Finally, a study of high-volume circumcision surgery at a fixed facility in Orange Farm, South Africa found that the procedure could be performed for an average of US$40, and that the procedure required 20 minutes (versus 29.5 for combined APHIA and NRHS at base facilities) including 7.5 minutes of the surgeon’s time (versus 16.3 for combined APHIA and NRHS). The reported cost was similar to the $38.33 and $39.58 per procedure we found for APHIA-II and NRHS, respectively [14]. However, these costs are not directly comparable since the Orange Farm estimate includes direct services and the rental and maintenance of the surgical space only. It excludes the cost of training, outreach and of overhead and administration. On the other hand, medical personnel salaries are higher in South Africa than in Kenya, and adjusting for this difference would move the unit cost estimates toward convergence. The shorter periods of total time for the procedure and for the surgeon’s time in the Orange Farm facility may be due to the different organization of the surgery including the use of disposable kits, and electrocautery which saves suturing time, the single most time-intensive part of the procedure. Another important study of MC costs in Zambia [20] was used as the basis of an MC scale-up modeling exercise carried out by the Futures Institute [4]. The data from Zambia suggests a unit cost of US$46.82, somewhat higher than the findings we report for Nyanza.

The observed variation in unit costs for these MC programs in Nyanza must also be considered in the context of far wider unit cost variation observed previously in HIV prevention programs. In our five-country study of 215 HIV prevention programs (the Prevent AIDS Network for Cost-Effectiveness Analysis [PANCEA] Project), we found variations in unit cost of 10- to 100-fold within a range of prevention strategies and countries [21]. These differences mainly represented variations in the number of delivered units of service, accompanied by some variation in the intensity of service per client, with relatively fixed personnel and other input costs. By comparison, the differences between adult MC approaches in Nyanza are small and are largely explained by variations in salaries. The roughly similar cost may reflect multiple homogenizing factors, including standardization of the service content; communication and coordination among the MC partners; and similar motivations by both APHIA II and NRHS program managers to try to optimize performance.

Further refinements in the staffing and logistical organization of the adult MC procedure itself may yield only modest gains in efficiency. This is because the marginal cost of supplies and personnel for each procedure is a small portion of the total unit cost. Yet, a dollar deducted from costs represents resources that can be freed to expand services, whatever the source of that savings. We therefore support further operations research into the possibility of streamlining the surgical procedure and immediately proximate activities. However, our data suggest that once programs have trained lower-cost surgical staff, large further reductions in costs must be sought elsewhere. The areas described in the accompanying Additional file 5 (Five Areas of Possible Efficiency Improvements for MC Delivery) include reduced staff wait times, trimmed overhead and more efficient scheduling of surgery days. Of these, scheduling and administrative efficiencies appeared most likely to yield a substantial reduction in cost per MC and thus per HIA. Operational efficiency (reducing start-up time on MC days) appears to offer smaller gains. Gains in technical efficiency through the Shang Ring and electro-cautery appear unavailable given their current costs and the relatively low cost of the labor and supplies they displace.

The measure of increased efficiency should be placed in this broader context of cost-effectiveness, using the cost per HIA metric, as it takes into account the possible trade-offs between potential economies and the number of MCs delivered.

### Study limitations

This report is limited to retrospective data from 222 MC service delivery sites over an 18-month period and prospective T&M data from 246 procedures. While these are sufficient to document unit costs and their variations, they are insufficient to support a robust multivariate analytic approach that might more definitively identifies the correlates of efficiency. Further, NRHS costing data for outreach and mobile services were unavailable in a disaggregated form, making only rough comparisons between the NRHS and APHIA approaches for outreach and mobile service delivery modes possible. The results might be quite different if mobile and outreach modes were separated for the NRHS approach. The T&M data were limited to a two-month period, and it is possible that seasonal variations in caseload or other factors captured by longer data collection period could affect the estimates of incremental per-case costs. The unit costs we reported are associated with the caseload shown in Figure 5. Since HIV intervention programs display economies of scale [21-23], if demand declines, unit costs could rise. This could occur if, for example, the most willing clients having already been served, and more money is needed for outreach to maintain the caseload size. Finally, we did not have data on the incidence of adverse events, though the cost of treating adverse events is implicitly captured in the personnel time and cost calculations.

The observed unit costs for adult MC programs in Nyanza, while likely to be similar in other provinces in Kenya, must also be considered in the context of far wider unit cost variation observed previously in the region. In particular the reported personnel costs are most relevant to other countries in which there is a clinical officer cadre. Personnel costs will be different in countries in which medical officers are required to be part of MC surgery. Moreover, MC programs using techniques other than the forceps-guided method may have different unit costs. The findings of this study, while not directly generalizable to other countries where MC programs are being implemented, offer insights into expected efficiencies and cost-effectiveness where similar types of program approaches and service delivery modes are being implemented.

## Conclusions

This study provides substantial evidence of similar efficiency for the various approaches of the Government of Kenya’s scale-up of its national adult MC program in Nyanza Province. Differences in unit cost between APHIA II and NRHS are modest, not consistently in the same direction, and to a large extent explained by differences in compensation levels. Thus, the observed differences in cost do not suggest that one approach is inherently more efficient than the other. The MC unit costs correspond to an estimated cost per HIV infection averted of $117 to $185, far less than the estimated $12,000 lifetime cost of treating HIV disease assuming $772 per person-year [22] for 22 years of treatment [24].

## Competing interests

The authors declare that they have no competing interests.

## Authors’ contributions

EAM led the process of research design, data analysis and interpretation, and the manuscript drafting and revision effort. SB coordinated data collection assisted with cleaning, data analysis and interpretation, manuscript drafting and editing. PP and JGK participated in research design, data analysis and interpretation, manuscript drafting and editing. All authors read and approved the final manuscript.

## Pre-publication history

The pre-publication history for this paper can be accessed here:

http://www.biomedcentral.com/1472-6963/14/31/prepub

## Supplementary Material

Additional file 1 Detail on allocation of costs to MC. Click here for file

Additional file 2 Percentage distribution of cost per procedure across cost components, by agency and delivery mode. Click here for file

Additional file 3 T-Test results - comparisons of time per MC procedure (minutes). Click here for file

Additional file 4 Differences in time per MC (minutes) by Approach and Service Delivery Mode. Click here for file

Additional file 5 Appendix - Efficiency improvements for MC delivery. Click here for file
