# Supplementary material for: Adult male circumcision in Nyanza, Kenya at scale: the cost and efficiency of alternative service delivery modes
Source: BMC Health Serv Res. 2014 Jan 23;14:31. doi: 10.1186/1472-6963-14-31 (PMC3902184; doi:10.1186/1472-6963-14-31)
Supplement: Additional file 1 — Detail on allocation of costs to MC. [file 1472-6963-14-31-S1.docx]

**Detail on allocation of costs to MC activities**

- ***Allocation of direct services personnel costs.*** During May 2010, and using an Excel-based data collection instrument, to guide program manager interviews, we recorded the number of hours per week that members of each personnel category spent on MC activities. The total MC-related full-time equivalents (FTEs) were further allocated to base, outreach, or mobile activities based on managers’ best estimates of designated duties and the time required by mobile and outreach activities, including transport time. The respective portion of each FTE was multiplied by the average compensation rate of the associated staff category to derive the cost of direct service provision.
- ***Allocation of support services personnel costs.*** The FTEs of support staff members (e.g., pharmacists, clerical assistants, and accounts clerks) were tabulated from administrative records and program manager interviews. Administrative support functions at the APHIA II base hospitals were apportioned to MC on the basis of the percentage that MC constituted of total inpatient-equivalent days, using methods prescribed by *WHO-CHOICE* [[13](#_ENREF_13)] for establishing the resource equivalence of outpatient visits versus inpatient days. The allocation of support staff time to service delivery mode was made on the basis of managers’ best estimates.
- ***Allocation of indirect costs*.** Building maintenance, utilities, vehicle storage and maintenance, and other miscellaneous non-personnel support costs at the APHIA II base hospitals were allocated to MC delivery mode according to the WHO-CHOICE method described above. The incremental MC-related indirect costs at outreach sites and at the NRHS base sites were unavailable but are believed to be minimal. In the case of outreach facilities, MC services are episodic; in the case of the NRHS sites, most of the indirect support costs are centralized in NRHS.
