# Supplementary material for: Adult male circumcision in Nyanza, Kenya at scale: the cost and efficiency of alternative service delivery modes
Source: BMC Health Serv Res. 2014 Jan 23;14:31. doi: 10.1186/1472-6963-14-31 (PMC3902184; doi:10.1186/1472-6963-14-31)
Supplement: Additional file 2 — Percentage distribution of cost per procedure across cost components, by agency and delivery mode. [file 1472-6963-14-31-S2.docx]

**Additional file 2: Percentage distribution of cost per procedure across cost components, by agency and delivery mode**


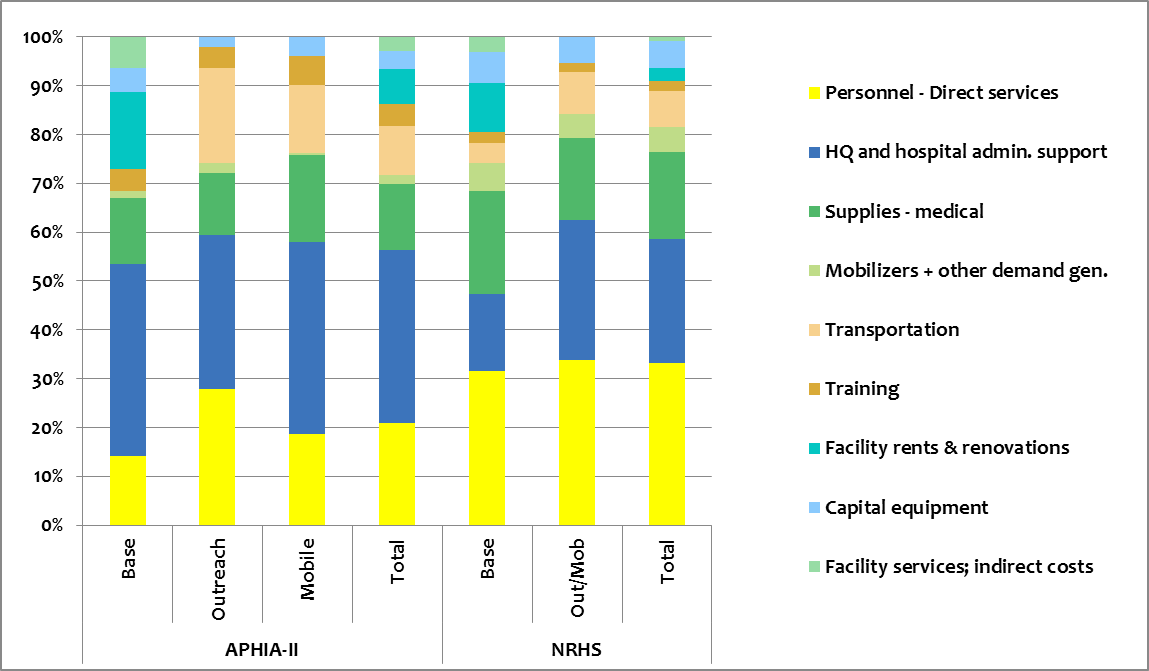


*Note:* Transportation includes fuel, maintenance, depreciation, insurance, and the value of staff time.
