# Supplementary material for: Adult male circumcision in Nyanza, Kenya at scale: the cost and efficiency of alternative service delivery modes
Source: BMC Health Serv Res. 2014 Jan 23;14:31. doi: 10.1186/1472-6963-14-31 (PMC3902184; doi:10.1186/1472-6963-14-31)
Supplement: Additional file 3 — T-Test results - comparisons of time per MC procedure (minutes). [file 1472-6963-14-31-S3.docx]

**Additional file 3: T-Test results - comparisons of time per MC procedure (minutes)**

Notes: MC steps continue from upper to lower block of rows. T-Test, 2-tailed for unequal variance. Highlight

signifies p-value < 0.05

Footnotes: 1. Surgeon time: "Marking" through "Sutures"; 2. Procedure time: "Placement on table" through "Dressing"; 3. Total time per case: "Procedure time" + transition time between dressing and post-op monitoring (not shown) + "Post-op time"; 4. Transition time on table: Sum of the transitions from "Injection of anaesthetic" through "Dressing". For economy of presentation, the individual transition times are not displayed in this table; 5. Post-op time: "Post-op monitoring", transition time, and "Instrument sterilization"; 6. Additional to any HIV C&T; 7. E.g., shaving; 8. Lay out instruments; other misc. surgical prep
