# Supplementary material for: Adult male circumcision in Nyanza, Kenya at scale: the cost and efficiency of alternative service delivery modes
Source: BMC Health Serv Res. 2014 Jan 23;14:31. doi: 10.1186/1472-6963-14-31 (PMC3902184; doi:10.1186/1472-6963-14-31)
Supplement: Additional file 4 — Differences in time per MC (minutes) by Approach and Service Delivery Mode. [file 1472-6963-14-31-S4.docx]

**Additional file 4: Differences in time per MC (minutes) by Approach and Service Delivery Mode**

Note: MC steps continue from upper to lower block of rows. Highlight signifies T-Test p-value < 0.05

1. Positive values signifies APHIA-II requires more time.

2. Positive values signifies "Base" requires more time.

3. Positive values signifies "Outreach" requires more time.

MCs performed by the NRHS program took, on average, 6.1 minutes less time than those performed by APHIA II including 2.7 minutes more of the surgeon’ time. MCs performed at base facilities required 4.8 minutes more of the surgeon’s time than those performed at mobile sites (p<0.05); and 7.5 minutes more per case. However, there were no statistically significant differences in the time required for the procedure at base facilities compared to outreach sites in either approach. Finally, in a comparison between mobile and outreach approaches, 9.5 minutes more were required per case at the outreach sites, of which 4.4 minutes were surgeons’ time.
