# Supplementary material for: Adult male circumcision in Nyanza, Kenya at scale: the cost and efficiency of alternative service delivery modes
Source: BMC Health Serv Res. 2014 Jan 23;14:31. doi: 10.1186/1472-6963-14-31 (PMC3902184; doi:10.1186/1472-6963-14-31)
Supplement: Additional file 5 — Appendix - Efficiency improvements for MC delivery. [file 1472-6963-14-31-S5.docx]

**Appendix**

**Five areas of Possible Efficiency Improvements for MC Delivery**

Through examination of the cost structure of MC delivery, we identified five potential strategies for improving efficiency. From a health care payer perspective, we explored the potential effects of these strategies on cost, HIV infections averted, and cost-effectiveness, using specified assumptions. The five strategies are: scheduling efficiency, operational efficiency, administrative efficiency, technical efficiency and demand generation:

***Scheduling efficiency:*** The mean number of MCs per day is far less than team capacity. Often, the same MC team visits one location on two consecutive days. If the number of MC-days were reduced by 50%, the clients seen in two days could potentially come on one day. However, it is likely that some clients would not be available on the one surgery day now available. We therefore assume a 10% lower demand.

***Operational efficiency:*** Waiting time prior to the first surgery of the day is similar to the time spent delivering the MC procedure. If the period prior to surgery was reduced by half, with no consequences on the number of MCs performed, staff time savings could be captured by reducing staff compensation or by having them perform other tasks.

***Administrative efficiency:*** Administrative costs represent 15–40% of total costs. We explored the potential effect on total MC costs of reducing administration by half. These savings would be realized by reducing office staffing—hiring fewer individuals, or reducing the portion of their time dedicated to MC programming. We assume no adverse effects on program quality or service delivery capacity.

***Technical efficiency—MC devices:*** New MC devices, such as the Shang Ring, can reduce the procedure duration and are simple to use. The Shang Ring procedure and removal requires 4.8 and 3.9 minutes, respectively, compared with 25.1 minutes for the standard forceps-guided procedure fund our time and motion observations.[[1](#_ENREF_1)] The Shang Ring requires no sutures and thus reduces the need for supplies and investment in surgical clamps. The most recent negotiated manufacturer’s price for the version of the Shang Ring currently being used in research studies in Kenya and Zambia is $9.

***Technical efficiency—electrocautery:*** Bleeding is often caused by transection of small blood vessels during foreskin removal. It can take several minutes to tie off these vessels and achieve hemostasis. Electrocautery is a quicker alternative to stitching, saving an estimated 4 minutes per MC, (personal communication from Emma Llewellyn, Director - Male Circumcision Project Nyanza Reproductive Health Society Kisumu, Kenya). Based on the use of electrocautery by NRHS-supported sites in Nyanza, we estimated that the reduced use of sutures and decreased wear on forceps saves an additional estimated $0.39. The electrocautery equipment, including coagulators, hand pieces, foot pedals, and electrodes, cost an estimated $2.00 per MC.

***Demand generation:*** Relatively little is spent in either the APHIA II or the NRHS approaches on mobilization to persuade men to seek MC. It is possible that more caseloads could be increased with a concerted marketing effort. Absent data on the variation of the cost of demand generation at various scales, we portray a variety of assumptions, including added cost per MC of $2.50 or of $5.00, increases in the number of MCs of 20% and 100%, and administrative costs either unaffected or partially affected by the increased number of MCs.

***Potential Efficiencies in MC Service Delivery***

**Cost savings per MC**

Table 1A shows the potential savings per MC, for each of the five strategies we assessed. *Administrative efficiency* has the largest potential savings. If administrative costs could be halved, the savings would total $3–$7.50 per MC, or 15–20% of total costs. *Scheduling efficiency* offers the next greatest potential savings, 8–20%. *Operational efficiency* offers smaller savings. The potential *technical efficiency* of Shang Ring and electro-cautery appears to be negative; they increase costs. The Shang Ring is likely to increased costs by $7.49 because the cost of the ring exceeds the value of saved supplies and personnel. Electro-cautery similarly adds more costs than the relatively modest reduction in time and supplies. For details and sensitivity analyses, see technical note below. Assuming a $5 cost per MC, 20% more MCs, and fixed administrative costs, *Demand generation* can save or add costs, depending on the service delivery mode. Savings occur when spreading fixed costs saves more than its $5. We conservatively assume no savings in the unit cost of supplies due to increased scale.

**Table 1 about here**

**Savings or Added Costs for Total MC Spending**

In Table 1B, the savings per MC are translated into total savings (or added costs) against a hypothetical standardized budget of $1 million However, for *demand generation*, a 20% increase in the number of procedures means that even with modest savings per MC, total costs increase.

**HIV infections averted (HIA)**

Table 1C shows the change in HIAs with each of the five strategies. This derives directly from the change in the number of MCs delivered. *Operational efficiency* and *Administrative efficiency* do not affect the number of MCs or HIAs. *Scheduling efficiency* is expected to decrease the number of infections averted, because lowering the number of MC days may mean that some clients will be unable to attend services. In contrast, *demand generation* increases MCs and HIAs, reflecting an assumption of 20% growth in demand for and provision of MCs.

**Cost per HIA**

Table 1D summarizes the incremental cost-effectiveness of the two strategies that affect HIAs. With *demand generation*, assuming an added cost of $5 per MC, 20% more MCs, and unaltered administrative costs, the estimated cost per HIA varies from $133 to $166, slightly more favorable than the cost-effectiveness ratios for the baseline MC program approaches.

*Scheduling efficiency* saves money but reduces the number of MCs. Thus, the cost-effectiveness ratio in Table 1D is the cost per HIA of *not* adopting this strategy. The values are higher than the baseline cost-effectiveness ratios for MC (from Table 7). Assuming locations with unmet demand, this suggests that it would be more efficient to do more MCs in other locations than to return to the same site on two consecutive days or to generate demand.

**Strategies for Increasing Efficiency**

Of the five strategies we examined, scheduling and administrative efficiencies appeared most likely to yield a substantial reduction in cost per MC and thus per HIA. Operational efficiency (reducing start-up time on MC days) appears to offer smaller gains. Gains in technical efficiency through the Shang Ring and electro-cautery appear unavailable given their current costs and the relatively low cost of the labor and supplies they displace.

Our study suggests that there is significant underutilized capacity to deliver MC services within current program operations. To take one example, travel costs, including foregone staff time, can be thought of as a fixed cost for a day of field-based services. The overall efficiency per surgery-day is then largely determined by how many cases can be completed during that day. Any increases from the current levels thus represent important efficiency gains. Since demand generation activities are currently a small portion of total costs, and since compensation and incentives to community mobilizers tend to be low (e.g., 750 Kenyan shillings per day), it may be feasible to increase uptake per surgery-day at a greater rate than the concomitant increased mobilization costs contribute to the cost per MC. Similarly, by decreasing the number of surgery-days at the same outreach or mobile sites, it may be possible to decrease transportation and daily start-up costs such that freed resources can fund expansion of MC activities elsewhere. These suggestions are particularly salient given our finding that efficiency represented by the time per procedure and the waiting time at the start of each surgery day is unrelated to the number of surgeries performed on the respective surgery days.

**Table 2 about here**

The table above, shows the cost-effectiveness associated with different input assumptions about demand generation. We vary three assumptions: the percentage rise in the number of MCs (20% or 100%); the increase in the cost of mobilization ($5 or $2.50) per MC; and how administrative costs are affected (fully fixed or 50% fixed/50% variable). A one hundred percent increase in MCs is an estimate of manageable daily MC procedures given the current team configuration. The lowest (most favorable) cost-effectiveness ratio, $46–66 per HIA, would be seen if demand generation doubled the number of MCs with fixed administrative costs (scenario c). Increasing the cost of mobilization (i.e., added cost per MC of $5 vs. $2.50) appears to have a greater effect on the cost per HIA than increased administrative costs (i.e., fixed vs. semi-fixed).

**Table 1: Cost savings per MC from strategies intended to increase MC delivery efficiency based on a hypothetical program with a $1 million annual budget**

| **A. Savings per MC (adjusted for change in no. of MCs)** | | | | | | | | | | | | | | | |
| --- | --- | --- | --- | --- | --- | --- | --- | --- | --- | --- | --- | --- | --- | --- | --- |
|  | **APHIA II** | | | | | | | | | | **NRHS** | | | | |
| **Strategy** | **Base** | | | | | **Outreach** | | | **Mobile** | | **Base** | | | **Outreach/ mobile** | |
| 1) Scheduling efficiency | $2.61 | | | | | $8.30 | | | $4.29 | | $3.30 | | | $5.80 | |
| 2) Operational efficiency | $1.00 | | | | | $3.35 | | | $1.10 | | $2.74 | | | $2.93 | |
| 3) Administrative efficiency | $7.53 | | | | | $6.36 | | | $5.71 | | $3.12 | | | $6.65 | |
| 4a) Technical efficiency—MC device | -$7.35 | | | | | -$6.40 | | | -$7.35 | | -$6.21 | | | -$5.72 | |
| 4b) Technical efficiency—electrocautery | -$1.22 | | | | | -$0.79 | | | -$1.22 | | -$0.48 | | | -$0.30 | |
| 5) Demand generation* | $0.85 | | | | | $0.67 | | | –$0.64 | | –$0.12 | | | $0.80 | |
| **B. Savings (added costs) for total MC spending** | | | | | | | | | | | | | | | |
|  | **APHIA II** | | | | | | | | | | | **NRHS** | | | |
| **Strategy** | **Base** | | | **Outreach** | | | | **Mobile** | | | | **Base** | | | **Outreach/ mobile** |
| 1) Scheduling efficiency | $161,351 | | | $284,473 | | | | $231,568 | | | | $174,995 | | | $213,064 |
| 2) Operational efficiency | $26,028 | | | $82,766 | | | | $37,412 | | | | $69,350 | | | $63,438 |
| 3) Administrative efficiency | $196,558 | | | $156,929 | | | | $194,892 | | | | $78,874 | | | $143,908 |
| 4a) Technical efficiency—MC device | -$191,806 | | | -$158,093 | | | | -$250,611 | | | | -$156,980 | | | $123,734 |
| 4b) Technical efficiency—electrocautery | -$31,723 | | | -$19,396 | | | | -$41,515 | | | | -$12,006 | | | -$6,396 |
| 5) Demand generation* | ($**173,275)** | | | ($**180,229)** | | | | ($**226,263)** | | | | ($**203,606)** | | | ($**179,134)** |
| **C. Effect (change in HIV infections averted) on HIAs of five strategies intended to increase**  **efficiency of MC service provision** | | | | | | | | | | | | | | | |
|  | **APHIA II** | | | | | | | | | | | **NRHS** | | | |
| **Strategy** | **Base** | | **Outreach** | | | | **Mobile** | | | | | **Base** | | **Outreach/ mobile** | |
| 1) Scheduling efficiency | (652) | | (617) | | | | (853) | | | | | (632) | | (541) | |
| 2) Operational efficiency | – | | – | | | | – | | | | | – | | – | |
| 3) Administrative efficiency | – | | – | | | | – | | | | | – | | – | |
| 4a) Technical efficiency—MC device | – | | – | | | | – | | | | | – | | – | |
| 4b) Technical efficiency—electrocautery | – | | – | | | | – | | | | | – | | – | |
| 5) Demand generation* | 1,305 | | 1,234 | | | | 1,705 | | | | | 1,263 | | 1,082 | |
| **D. Cost per HIV infection averted^1^ on cost per HIA of scheduling efficiency and demand generation to increase efficiency of MC service provision** | | | | | | | | | | | | | | | |
| **Strategy** | | **APHIA II** | | | | | | | | | | **NRHS** | | | |
|  |  | **Base** | | | **Outreach** | | | | | **Mobile** | | **Base** | **Outreach/ mobile** | | |
| 1) Scheduling efficiency (strategy-->baseline) | | $247 | | | $461 | | | | | $272 | | $277 | $394 | | |
| 3) Administrative efficiency | | n/a | | | n/a | | | | | n/a | | n/a | n/a | | |
| 4a) Technical efficiency—MC device | | n/a | | | n/a | | | | | n/a | | n/a | n/a | | |
| 4b) Technical efficiency—electrocautery | | n/a | | | n/a | | | | | n/a | | n/a | n/a | | |
| 5) Demand generation* | | $133 | | | $146 | | | | | $133 | | $161 | $166 | | |

**Note:* Assumptions for demand generation are: $5 per MC, 20% more MCs, and fixed administrative costs)

1. Costs are unadjusted for reduced HIV care costs

**Table 2: Sensitivity analysis: Effect of demand generation on cost per HIA (cost-effectiveness), by value of input assumptions**

| **Demand generation: cost per HIV infection averted (unadjusted for HIV care costs)** | | | | | |
| --- | --- | --- | --- | --- | --- |
| **Rise in no. of MCs; mobilization cost per case; admin. costs fixed or semi-fixed** | **APHIA II** | | | **NRHS** | |
|  | **Base** | **Outreach** | **Mobile** | **Base** | **Outreach/ mobile** |
| a. 20%; $5; fixed | $133 | $146 | $133 | $161 | $166 |
| b. 20%; $5; semi-fixed | $160 | $169 | $153 | $173 | $190 |
| c. 100%; $5; fixed | $46 | $53 | $46 | $66 | $67 |
| d. 100%; $5; semi-fixed | $67 | $70 | $62 | $75 | $85 |
| e. 20%; $2.50; fixed | $83 | $96 | $83 | $111 | $116 |
| f. 20%; $2.50; semi-fixed | $110 | $119 | $103 | $123 | $140 |

**Technical Note**

**Calculation of the incremental costs resulting from the adoption of the Shang Ring and the Electro-Cautery**

**1. Shang Ring.**

The Shang Ring obviates the need for certain durable equipment items, for suture and for a portion of the surgeon’s time. These changes save money; but the Shang Ring itself entails a significant new expense. We used data on the cost and quantity of the various inputs saved, and balanced these savings against the cost of the Shang Ring to determine whether the Shang Ring was likely to save money. Given the current cost of $9.00 per Shang Ring we found that it would generate net costs of $7.49 per procedure, and that the break-even cost of the Shang Ring in the context of the MC programs we studied in Nyansa, Kenya was $1.51.

These calculations were made with a spreadsheet-based model that calculated the incremental net cost of the Shang Ring based on the following inputs:

The cost of the items eliminated including Allis, “mosquito”, artery and dissection forceps, needle holders and suture scissors is $49.61. We assumed that on average, these items can be used for 1,000 procedures before replacement. The cost of the chromic catgut 3/0 suture replaced is $0.73. Personnel cost savings estimates were based on a reduction in average conventional procedure time which is 25.1 minutes. From this we deducted the Shang Ring procedure and device removal times of 4.8 and 3.9 minutes, respectively, (Barone MA, Ndede F, Li PS, Masson P, Awori Q, et al. (2011) *The Shang Ring device for adult male circumcision: a proof of concept study in Kenya.* J Acquir Immune Defic Syndr 57: e7-12). The time saved was then multiplied by the compensation rate of a surgical nurse. The calculation is summarized in the Table below.

**Savings, additional costs and net costs associated with the Shang Ring compared with the conventional forceps guided procedure.**

| **Cost component** | **Savings per MC** | **Additional cost**  **per MC** |
| --- | --- | --- |
| Equipment | $0.05 |  |
| Disposable supplies | $0.73 | $9.00 |
| Personnel | $0.73 |  |
| Total savings and cost | $1.51 | $9.00 |
| **Net cost** | **$7.49** | |

The finding of significant net costs associated with the Shang Ring is robust to widely varied assumptions about uncertain input values. If equipment needed replacement after 100 procedures rather that 1,000 the net cost is $7.04. If the operator earned twice as much as in our base case calculation, and the time saved were double, the net cost would still be $5.29. The spreadsheet-based model on which the table above is based, is available on request from the authors.

**2. Electro-Cautery.**

We performed an analysis of the net costs associated with the electro-cautery structured similarly to that of the Shang Ring above: Incremental per-procedure costs and savings were tallied and compared. The result is shown in the table below under the assumption that the cautery itself has a useful life of 1,000 procedures and the re-usable hand-pieces, foot pedals and electrodes have a life of 250 procedures. Equipment cost estimates were derived from pro-forma invoices provided by NRHS. Personnel cost savings assume a saving of 4 minutes per procedure (personal communication from Emma Llewellyn, Director - Male Circumcision Project Nyanza Reproductive Health Society Kisumu, Kenya to co-author, Paul Perchal).

**Savings, additional costs and net costs associated with the Electro-Cautery compared with the conventional suturing.**

| **Cost component** | **Savings per MC** | **Additional cost**  **per MC** |
| --- | --- | --- |
| Equipment-cautery |  | $1.02 |
| Equipment-hand pieces |  | $0.32 |
| Equipment-foot pedals |  | $0.32 |
| Equipment-electrodes |  | $0.35 |
| Mosquito forceps | $0.02 |  |
| Sutures | $0.37 |  |
| Personnel | $0.59 |  |
| Total savings and cost | $0.97 | $2.00 |
| **Net cost** | **$1.03** | |

If the useful life of both the cautery and its auxiliary equipment were doubled to 2,000 and 500 procedures respectively, the electro-cautery approaches break-even. Similarly, if the savings in personnel time were 12 minutes per procedure rather than 4, the electro-cautery approaches break-even. The spreadsheet-based model on which the table above is based, is available on request from the authors.

**References**

1. Barone MA, Ndede F, Li PS, Masson P, Awori Q, Okech J, Cherutich P, Muraguri N, Perchal P, Lee R *et al*: **The Shang Ring device for adult male circumcision: a proof of concept study in Kenya**. *Journal of acquired immune deficiency syndromes (1999)* 2011, **57**(1):e7-12.
